# Concerning trends in maternal risk factors in the United States: 1989–2018

**DOI:** 10.1016/j.eclinm.2020.100657

**Published:** 2020-11-20

**Authors:** Eran Bornstein, Yael Eliner, Frank A. Chervenak, Amos Grünebaum

**Affiliations:** a Division of Maternal-Fetal Medicine, Department of Obstetrics and Gynecology, Lenox Hill Hospital – Northwell Health/Zucker School of Medicine, New York, NY, United States; b Boston University, School of Public Health, Boston, MA, United States

**Keywords:** Pregnancy risk factors, Hypertensive disorders of pregnancy, Chronic hypertension, Diabetes mellitus, Advanced maternal age, Grand multiparity

## Abstract

**Background:**

Increased efforts have focused on reducing maternal morbidity and mortality in the United States (US). Hypertensive disorders of pregnancy, chronic hypertension, diabetes mellitus, very advanced maternal age, and grand multiparity are known contributors to various maternal morbidities, as well as maternal mortality. We aimed to evaluate the trends in these risk factors/complications among US pregnancies during the last three decades (1989–2018).

**Methods:**

This is a retrospective study based on the CDC natality database. We calculated the annual prevalence of each risk factor/complication from 1989 to 2018. Joinpoint regression analysis was then used to evaluate the trends. Annual percentage changes (APC) were calculated for each of the segments identified by the joinpoint regression, and average annual percentage changes (AAPC) were calculated for the entire period. Relative risks (RR) comparing the prevalence of each risk factor/complication in 2018 to its prevalence in 1989 were also calculated. Subsequent analyses evaluated the trends of the main risk factors/complications by maternal age groups. Statistical significance was determined at *p*<0·05, and results were presented with 95% confidence intervals.

**Findings:**

Between 1989 and 2018, the prevalence of hypertensive disorders of pregnancy increased by 149% (AAPC 3·2, 95% CI 2·6–3·8), that of chronic hypertension increased by 182% (AAPC 3·7, 95% CI 3·3–4·2), that of diabetes mellitus increased by 261% (AAPC 4·6, 95% CI 4·0–5·2), that of very advanced maternal age increased by 194% (AAPC 3·8, 95% CI 3·6–4·0), and that of grand multiparity increased by 33% (AAPC 1·0, 95% CI 0·8–1·2). Chronic hypertension and diabetes mellitus increased mostly during the past two decades, while hypertensive disorders of pregnancy and grand multiparity increased primarily over the most recent decade. Additionally, women of very advanced maternal age had significantly higher rates of hypertensive disorders of pregnancy, chronic hypertension and diabetes mellitus throughout our study period.

**Interpretation:**

Our study shows a marked increase in the prevalence of five pregnancy risk factors/complications over the past three decades (1989–2018). This may point to a significant deterioration in the health of US pregnant women, which potentially contributes to both maternal morbidity and mortality.

**Funding:**

None.

**Research in Context Panel**

**Evidence before this study**

Prior studies have documented increasing rates of hypertensive disorders of pregnancy, chronic hypertension, diabetes mellitus, very advanced maternal age, and grand multiparity among pregnant women in the United States (US). These studies were typically limited by either the size of the sampled population or the length of the time period examined, as well as by the statistical method used to evaluate temporal trends. We searched PubMed for any article published up to March 1, 2020, referring to the prevalence and trends in hypertensive disorders of pregnancy / pregnancy induced hypertension / gestational hypertension / preeclampsia / eclampsia / chronic hypertension / diabetes mellitus / pre-gestational diabetes / gestational diabetes / advanced maternal age / maternal age / grand multiparity and parity.

**Added value of this study**

We document a marked increase in the prevalence of several maternal risk factors/complications: hypertensive disorders of pregnancy, chronic hypertension, diabetes mellitus, very advanced maternal age, and grand multiparity among the entire population of US pregnant women over the last 30 years (1989 to 2018). The added value of this study stems from evaluating these trends on the entire population of US pregnant women and over such an extended period of time. In addition, by conducting joinpoint regression analyses, we are able to illustrate the temporal changes of the trends in each of these risk factors/complications throughout our 30 year study period.

**Implications of all the available evidence**

Our results point to a significant and continuous deterioration in the health of US pregnant women over the past three decades, which has been especially prominent during the last decade. Understanding the trends in maternal risk factors/complications could help identify the potential root causes for the increasing rates of maternal morbidity and mortality in the US, as well as target intervention measures to curtail these trends.

Alt-text: Unlabelled box

## 1 Introduction

Maternal mortality rates in the United States (US) have more than doubled during the past three decades, increasing from 7·9 per 100,000 live births in 1989 to 17·4 per 100,000 live births in 2018 [1,2]. Significant increases have also been documented throughout the years in the prevalence of indicators for severe maternal morbidities and pregnancy complications, such as acute renal failure, pulmonary embolism, adult respiratory distress syndrome (ARDS), acute myocardial infarction, aneurysm, shock, blood transfusion, hysterectomy, and ventilation [3], [4], [5]. It was estimated that severe maternal morbidities affected more than 50,000 US women in 2014 [5].

The reasons for the increasing trends in maternal morbidity and mortality are not entirely clear. Changes in the pre-conception health of pregnant women, trends of delaying childbearing, increased rates of cesarean deliveries [6], as well as racial and ethnic disparities [7], have been suggested as major contributors. Improvements in the level of surveillance and identification of pregnancy related morbidities and mortalities have also played an important role [2,8,9].

In this study, we aim to extend prior research and evaluate the trends in several major pregnancy risk factors and complications – hypertensive disorders of pregnancy (HDP), chronic hypertension (CH), diabetes mellitus (DM), very advanced maternal age (vAMA), and grand multiparity (GM) – among US gravidas during the three decade period between 1989 and 2018. Understanding the long-term trends in these risk factors/complications can potentially help clarify the underlying reasons for the increasing rates of maternal morbidity and mortality in the US during this time period [10].

## 2 Methods

### 2.1 Data and risk factor definitions

This is a retrospective cohort study for the 30 year period from 1989 to 2018. Data regarding the annual cases of live births complicated by hypertensive disorders of pregnancy, chronic hypertension, diabetes mellitus, very advanced maternal age, and grand multiparity, as well as the total number of live births per year, were collected from the National Center for Health Statistics’ natality files. The data for 1995–2018 were accessed through the Center for Disease Control and Prevention's (CDC) Wonder system [11]. The data for 1989–1994 were collected from the National Vital Statistics Reports [12]. For parity, the Wonder system only included data since 2003 [11], and data for all prior years were collected from the National Vital Statistics Reports [12]. The data include all live births occurring to US residents within the United States.

Data on hypertensive disorders of pregnancy (HDP; defined as gestational hypertension, mild and severe preeclampsia, eclampsia, and HELLP syndrome), chronic hypertension (CH), and diabetes mellitus (DM; includes pre-gestational type I diabetes, pre-gestational type II diabetes, and gestational diabetes) are available in the natality files since 1989, following the implementation of the 1989 revision of the US Standard Certificate of Live Birth. These data remain comparable throughout the 30-year period until 2018. Very advanced maternal age (vAMA) was identified from data on the age group of the mother and defined as women who were 40 years or older at the time of delivery. Grand multiparity (GM) was identified from detailed parity data and defined as women with at least five live births. For each risk factor/complication, incomplete records were excluded.

### 2.2 Statistical analyses

For every year between 1989 and 2018, we calculated the annual prevalence of HDP, CH, DM, vAMA, and GM as percentages of live births. Subsequently, we evaluated the temporal trends in each pregnancy risk factor/complication by way of joinpoint regression analysis, conducted using the Joinpoint Regression Program, Version 4·8·0·1, by the National Cancer Institute. The joinpoint regression method is used to detect changes in data trends. In our application, it was set to fit a model of connected straight lines to a logarithmic transformation of the data. For each potential number of joinpoints (i.e. points in which the trend changes) between 0 and 9, the locations of the joinpoints were determined via the grid method, by creating a grid of all possible joinpoint locations, fitting a model to each possibility, and choosing the model with the lowest sum of squared errors (SSE) [13]. Then, the best model, with the optimal number of joinpoints, was chosen by comparing the models with the different number of joinpoints and testing whether more joinpoints were statistically significant. Statistical significance was tested via the permutation method, by running 10,000 Monte Carlo simulations [14]. The reported results of the model include the locations of the joinpoints, the annual percentage change (APC) for each segment of the trend (equal to the year-over-year change in the rate, under the assumption that this change is constant for the segment), and the average annual percentage change (AAPC) for the full time period (equal to a weighted average of all of the APCs). In addition, as an alternative method, Pearson chi-square testing was used to compare the prevalence of each risk factor/complication in 2018 to its prevalence in 1989, with results presented as relative risks (RR). This analysis was conducted using SAS, Version 9·4. In subsequent analyses, we calculated the proportion of pregnancies in each of six maternal age groups (under 20 years, 20–24 years, 25–29 years, 30–34 years, 35–39 years, and at or above 40 years of age), as well as the prevalence of HDP, CH, and DM among these age groups, for every year between 1989 and 2018. For every age group, the temporal trends of HDP, CH, and DM were then evaluated by way of the joinpoint regression method, as well as by calculating relative risks.

For all tests, statistical significance was determined as a p-value below 0·05, and results were reported with associated 95% confidence intervals (95% CI).

### 2.3 Ethics

Institutional review board approval and informed consent were not required because the anonymized data are publicly available through a data use agreement with the National Center for Health Statistics.

### 2.4 Role of the funding source

No funding was obtained for this study.

## 3 Results

From 1989 to 2018, there were a total of 120,705,674 live births in the US, with an average of 4023,522 live births per year (ranging from 3791,712 in 2018 to 4316,233 in 2007). Of the births with available data, 4·09% were to women with hypertensive disorders of pregnancy (HDP), 1·07% were to women with chronic hypertension (CH), 4·09% were to women with diabetes mellitus (DM), 2·39% were to women 40 years of age or more (vAMA), and 4·5% were to grand multiparous women (GM).

**Fig. 1:**
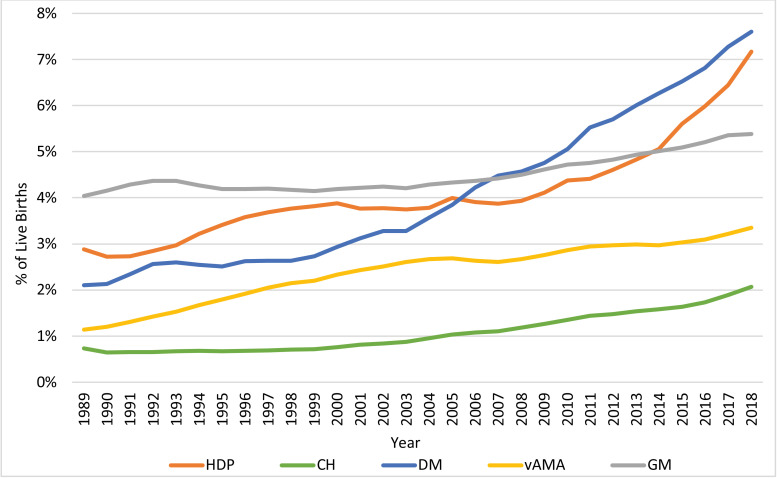
Trends in the Prevalence of the Pregnancy Risk Factors/Complications: 1989–2018. The figure presents the annual proportions of live births attributed to US women with hypertensive disorders of pregnancy (HDP), chronic hypertension (CH), diabetes mellitus (DM), very advanced maternal age (vAMA), and grand multiparity (GM).

**Fig. 2:**
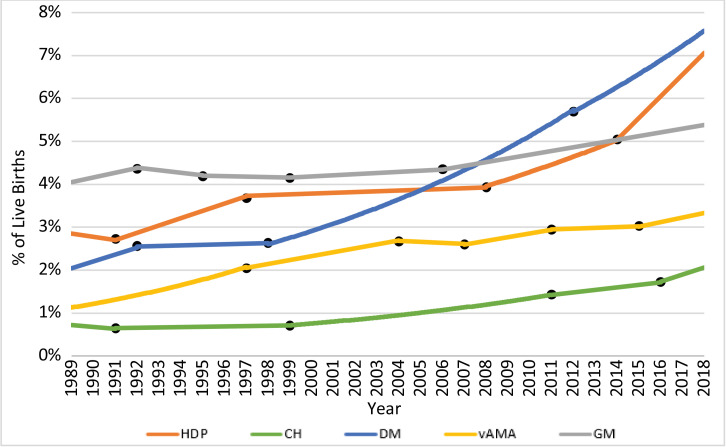
Joinpoint Regression Analysis of the Trends in the Prevalence of the Pregnancy Risk Factors/Complications: 1989–2018 – Graphical Presentation. The figure presents the trends in the prevalence of hypertensive disorders of pregnancy (HDP), chronic hypertension (CH), diabetes mellitus (DM), very advanced maternal age (vAMA), and grand multiparity (GM) among US gravidas, as analyzed via the joinpoint regression method. The dots mark the joinpoints identified by the joinpoint regression, and the lines between the dots depict the trend in each segment.

The temporal trends in the prevalence of each of the risk factors/complications from 1989 to 2018 are displayed in Fig. 1. The segmentation results of the joinpoint regression analyses are presented in Fig. 2, with dots marking the joinpoints, and the detailed joinpoint results are presented in Table 1. Each risk factor/complication has a different number of joinpoints and therefore a different number of segments. Relative risks comparing the prevalence of each risk factor/complication in 2018 to its prevalence in 1989 are also presented in Table 1. During this 30-year period, the prevalence of HDP increased by 149% (RR 2·49, 95% CI 2·47–2·50, *p*<0·001) with an AAPC of 3·2 (95% CI 2·6–3·8, *p*<0·001), the prevalence of CH increased by 182% (RR 2·82, 95% CI 2·78–2·86, *p*<0·001) with an AAPC of 3·7 (95% CI 3·3–4·2, *p*<0·001), the prevalence of DM increased by 261% (RR 3·61, 95% CI 3·58–3·64, *p*<0·001) with an AAPC of 4·6 (95% CI 4·0–5·2, *p*<0·001), the prevalence of vAMA increased by 194% (RR 2·94, 95% CI 2·91–2·98, *p*<0·001) with an AAPC of 3·8 (95% CI 3·6–4·0, *p*<0·001), and the prevalence of GM increased by 33% (RR 1·33, 95% CI 1·32–1·34, *p*<0·001) with an AAPC of 1·0 (95% CI 0·8–1·2, *p*<0·001).

**Table 1:** Joinpoint Regression Analysis of the Trends in the Prevalence of the Pregnancy Risk Factors/Complications: 1989–2018 – Detailed Results The table presents the results of the joinpoint regression analyses for hypertensive disorders of pregnancy (HDP), chronic hypertension (CH), diabetes mellitus (DM), very advanced maternal age (vAMA), and grand multiparity (GM). Each risk factor/complication has a different number of joinpoints and therefore a different number of segments. For each segment, we report the annual percentage change (APC), and for the entire 1989–2018 period, we report the average annual percentage change (AAPC). Additionally, relative risks (RR) for the entire period are also presented.

	HDP	CH	DM	vAMA	GM
**Segment #1**	**1989–1991** APC −2·6 95% CI [−8·3,3·5] *p* = 0·375	**1989–1991** APC −5·0 95% CI [−9·7,0·0] *p* = 0·049	**1989–1992** APC 7·7 95% CI [3·2,12·5] *p* = 0·002	**1989–1997** APC 7·8 95% CI [7·6,8·1] *p*<0·001	**1989–1992** APC 2·8 95% CI [2·0,3·6] *p*<0·001
**Segment #2**	**1991–1997** APC 5·6 95% CI [4·3,6·9] *p*<0·001	**1991–1999** APC 1·2 95% CI [0·5,1·9] *p* = 0·002	**1992–1998** APC 0·3 95% CI [−1·4,2·1] *p* = 0·692	**1997–2004** APC 3·9 95% CI [3·6,4·2] *p*<0·001	**1992–1995** APC −1·4 95% CI [−3·0,0·1] *p* = 0·072
**Segment #3**	**1997–2008** APC 0·4 95% CI [0·0,0·8] *p* = 0·033	**1999–2011** APC 5·9 95% CI [5·6,6·2] *p*<0·001	**1998–2012** APC 5·8 95% CI [5·5,6·2] *p*<0·001	**2004–2007** APC −1·3 95% CI [−2·8,0·3] *p* = 0·096	**1995–1999** APC −0·3 95% CI [−1·1,0·5] *p* = 0·397
**Segment #4**	**2008–2014** APC 4·3 95% CI [3·3,5·4] *p*<0·001	**2011–2016** APC 3·8 95% CI [2·8,4·9] *p*<0·001	**2012–2018** APC 4·6 95% CI [3·8,5·5] *p*<0·001	**2007–2011** APC 3·2 95% CI [2·4,4·0] *p*<0·001	**1999–2006** APC 0·7 95% CI [0·4,0·9] *p*<0·001
**Segment #5**	**2014–2018** APC 8·8 95% CI [7·5,10·2] *p*<0·001	**2016–2018** APC 9·7 95% CI [6·5–13·1] *p*<0·001	··	**2011–2015** APC 0·5 95% CI [−0·2,1·3] *p* = 0·165	**2006–2018** APC 1·8 95% CI [1·7,1·9] *p*<0·001
**Segment #6**	··	··	··	**2015–2018** APC 3·5 95% CI [2·7,4·3] *p*<0·001	··
**1989–2018 AAPC**	AAPC 3·2 95% CI [2·6,3·8] *p*<0·001	AAPC 3·7 95% CI [3·3,4·2] *p*<0·001	AAPC 4·6 95% CI [4·0,5·2] *p*<0·001	AAPC 3·8 95% CI [3·6,4·0] *p*<0·001	AAPC 1·0 95% CI [0·8,1·2] *p*<0·001
**1989–2018 RR**	RR 2·49 95% CI [2·47–2·50] *p*<0·001	RR 2·82 95% CI [2·78–2·86] *p*<0·001	RR 3·61 95% CI [3·58–3·64] *p*<0·001	RR 2·94 95% CI [2·91–2·98] *p*<0·001	RR 1·33 95% CI [1·32–1·34] *p*<0·001

Notably, however, there were many changes in the behavior of the temporal trends throughout the years. The prevalence of HDP increased consistently in the 1990s, plateaued until 2008, and has increased rapidly since then, and especially since 2014 (2014–2018 APC: 8·8, *p*<0·001). The prevalence of CH increased only slightly during the 1990s, but has been increasing continuously since 1999, with an especially high APC since 2016 (2016–2018 APC: 9·7, *p*<0·001). The prevalence of DM increased significantly in the early 1990s, stabilized until 1998, and has since then been growing exponentially, albeit at a slightly lower APC since 2012 (2012–2018 APC: 4·6, *p*<0·001). The prevalence of vAMA increased consistently during the 1990s through 2004, with an especially high APC in the 1989–1997 period (APC: 7·8, *p*<0·001), and then stabilized and increased alternately, every 3–5 years, eventually continuing to increase since 2015 (2015–2018 APC: 3·5, *p*<0·001). The prevalence of GM increased between 1989 and 1992, then decreased slightly and stabilized, before continuing to increase between 1999 and 2018, with a higher APC since 2006 (2006–2018 APC: 1·8, *p*<0·001).

**Fig. 3:**
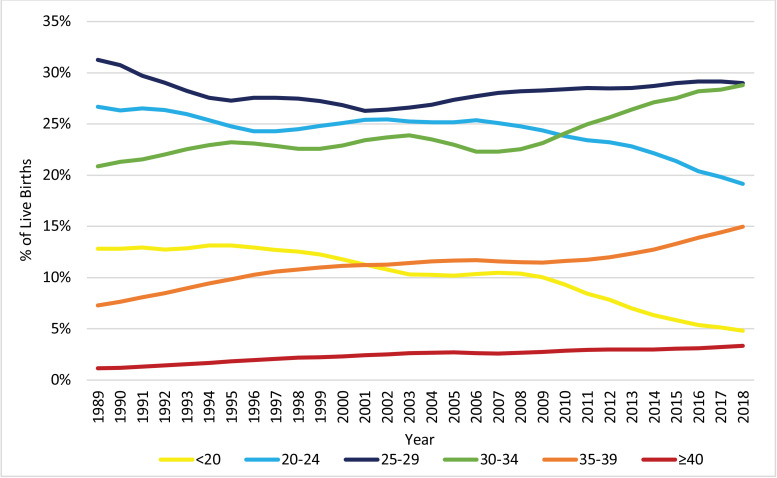
Trends in the Distribution of Live Births: 1989–2018, by Age Group (in Years). The figure presents the annual proportions of live births to US women in six age groups: under 20 years, 20–24 years, 25–29 years, 30–34 years, 35–39 years, and at or above 40 years of age.

**Fig. 4:**
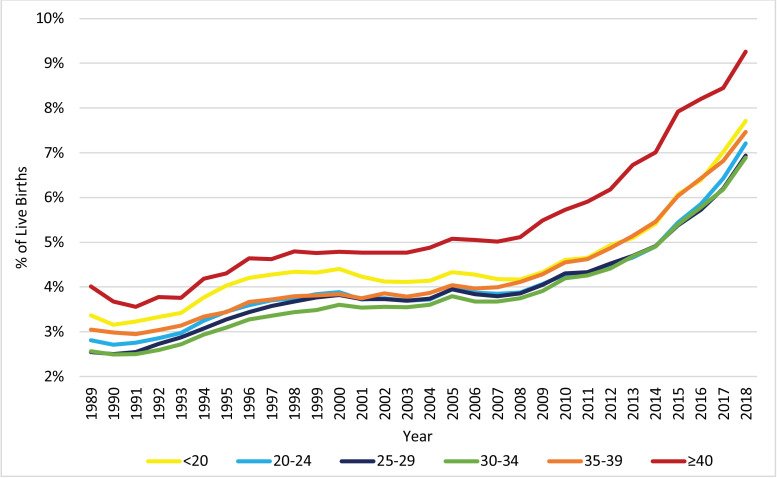
Trends in the Prevalence of HDP: 1989–2018, by Age Group (in Years). The figure presents the annual proportions of live births attributed to US women with hypertensive disorders of pregnancy (HDP) in six age groups: under 20 years, 20–24 years, 25–29 years, 30–34 years, 35–39 years, and at or above 40 years of age.

**Fig. 5:**
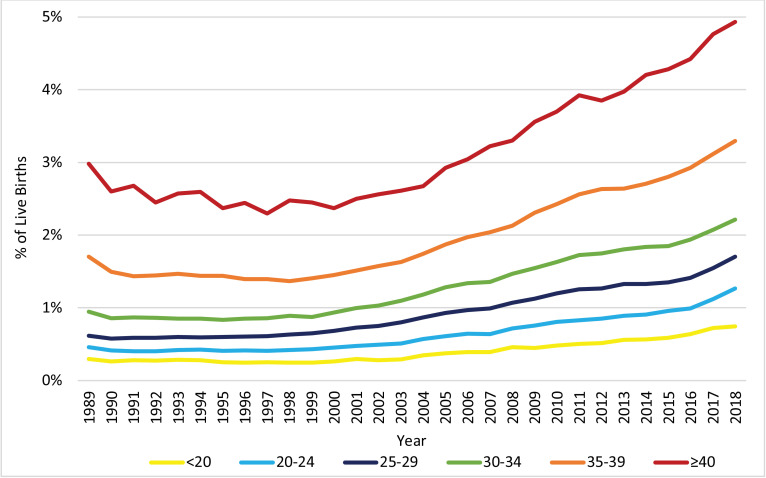
Trends in the Prevalence of CH: 1989–2018, by Age Group (in Years). The figure presents the annual proportions of live births attributed to US women with chronic hypertension (CH) in six age groups: under 20 years, 20–24 years, 25–29 years, 30–34 years, 35–39 years, and at or above 40 years of age.

**Fig. 6:**
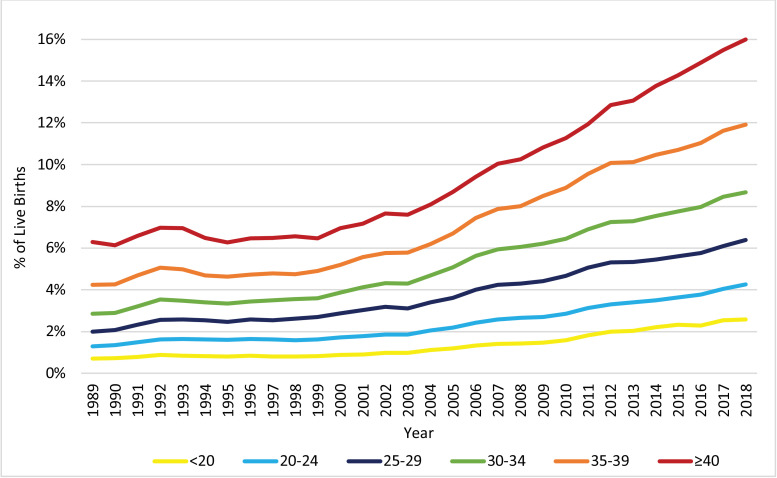
Trends in the Prevalence of DM: 1989–2018, by Age Group (in Years). The figure presents the annual proportions of live births attributed to US women with diabetes mellitus (DM) in six age groups: under 20 years, 20–24 years, 25–29 years, 30–34 years, 35–39 years, and at or above 40 years of age.

The distribution of live births across the age groups of the mother, as displayed in Fig. 3, changed significantly from 1989 to 2018. Notably, the proportion of pregnancies to women at or above the age of 30 increased, while the proportion of pregnancies to women below the age of 30 decreased. Fig. 4, Fig. 5, Fig. 6 display the trends in HDP, CH, and DM, respectively, stratified by age group. All age groups exhibited significant increases in the prevalence of HDP, CH, and DM between 1989 and 2018, but the prevalence of these risk factors/complications was consistently higher for women with vAMA compared to the other age groups.

The prevalence of HDP was highest for women at or above the age of 40. Before 2008, women under the age of 20 had the second highest prevalence, but since 2008, the gap between women in the <20 and 35–39 age groups has reduced. Overall, all age groups exhibited a similar increasing trend, with a dramatic increase in the prevalence of HDP since 2008 (Fig. 4). During the entire 1989–2018 period, women in the 25–29 and 30–34 age groups exhibited the largest relative increase in the prevalence of HDP (AAPC 3·5, 95% CI 2·9–4·1, *p*<0·001, with RR 2·72, 95% CI 2·68–2·76, *p*<0·001, and AAPC 3·5, 95% CI 2·9–4·0, *p*<0·001, with RR 2·69, 95% CI 2·65–2·73, *p*<0·001, respectively). Meanwhile, women in the <20 and ≥40 age groups exhibited the lowest relative increase in the prevalence of HDP (AAPC 2·9, 95% CI 2·2–3·6, with RR 2·29, 95% CI 2·24–2·34, and AAPC 2·9, 95% CI 1·9–3,9, with RR 2·30, 95% CI 2·20–2·42, respectively).

The prevalence of CH was consistently higher as the age group increased. Moreover, for all age groups, the prevalence of CH was relatively stable in the 1990s, with slight declines for the <20 and ≥40 age groups. Since the late 1990s/early 2000s, the prevalence of CH increased significantly among all age groups, but at varying APCs (Fig. 5). Notably, the relative increase in the prevalence of CH between 1989 and 2018, was substantially lower for the age groups of 35–39 (AAPC 2·4, 95% CI 1·9–2·8, *p*<0·001; RR 1·94, 95% CI 1·88–2·00, *p*<0·001) and ≥40 (AAPC 2·1, 95% CI 1·8–2·5, *p*<0·001; RR 1·66, 95% CI 1·56–1·76, *p*<0·001), than for the younger age groups (AAPCs of 3·0–3·7, *p*<0·001; RRs of 2·33–2·78, *p*<0·001).

The prevalence of DM was also consistently higher as the age group increased. Furthermore, for all age groups, after a fast increase in the beginning of the decade, the prevalence of DM was relatively stable during the rest of the 1990s, but has increased dramatically since the late 1990s/early 2000s (Fig. 6). From 1989 to 2018, the relative prevalence increase was substantially higher for the <20 age group (AAPC 4·9, 95% CI 4·1–5·7, *p*<0·001; RR 3·70, 95% CI 3·54–3·86, *p*<0·001) and substantially lower for the ≥40 age group (AAPC 3·1, 95% CI 2·8–3·5, *p*<0·001; RR 2·55, 95% CI 2·45–2·65, *p*<0·001), compared to the other groups (AAPCs of 3·7–4·2 and RRs of 2·81–3·28). It is noteworthy, however, that since 1999 the AAPC (calculated as a weighted average of the APCs during the partial period) for women in the ≥40 age group was slightly higher than those for women of age 25 to 39 (AAPC of 4·6 compared to AAPCs of 3·5–4·0), and that during the most recent decade (2008–2018) it was significantly higher than those for women of age 25 to 39 (AAPC of 4·6 compared to AAPCs of 3·5–4·0).

## 4 Discussion

Our study documents dramatic increases in the prevalence of several pregnancy risk factors/complications – HDP, CH, DM, vAMA, and GM – among US pregnant women during the last three decades. For CH and DM, these increases have primarily occurred since the late 1990s/early 2000s, and for HDP and GM, they were more pronounced since the mid/late 2000s. Although the trends in HDP, CH, and DM were observed in all age groups, vAMA women consistently had higher rates of these risk factors/complications compared to women in younger age groups. This is especially disturbing, as the proportion of pregnancies to vAMA women has also grown during this time period.

Our findings are consistent with several smaller studies that reported increases in the rates of HDP, CH, DM, vAMA, and GM among different samples of pregnant women in the US and during different time periods over the past three decades. For example, Kuklina et al. (2009) used the Nationwide Inpatient Sample of the Healthcare Cost and Utilization Project (which includes data from 20% of all nonfederal community hospitals in participating states) and found that the overall prevalence of all hypertensive disorders among delivery hospitalizations (including both HDP and CH) increased from 6·72% in 1998 to 8·34% in 2006 [3]. Using the same database, Bateman et al. (2012) focused specifically on CH and reported that the age-adjusted prevalence of CH increased from 1·01% in 1995–1996 to 1·76% in 2007–2008 [15]. With regard to DM, Lawrence et al. (2008) focused on a sample of women in Southern California during the period between 1999 and 2005 and documented increases in the prevalence of pre-existing diabetes from 0·76% to 1·9% and gestational diabetes from 7·1% to 7·8% [16]. Our results not only confirm these prior findings, but also illustrate the magnitude of the increases in the prevalence of these risk factors/complications among the entire US pregnant population. Our results further show that the increase in the prevalence of all risk factors/complications continued through 2018. In the last three years alone (2016–2018), we find a prevalence increase of 27·9% for HDP, 26·0% for CH, 16·6% for DM, 10·3% for vAMA, and 5·6% for GM. Moreover, by using one consistent and comparable dataset with 30 years of relevant data, we are able to document the long-term increasing temporal trends in HDP, CH, DM, vAMA, and GM between 1989 and 2018, as well as to illustrate the changes in these trends throughout the years.

The prevalence of HDP increased significantly from 2·88% in 1989 to 7·17% in 2018 (AAPC of 3·2 and RR of 2·49). After stabilizing in the late 1990s/early 2000s, the prevalence of HDP has increased dramatically over the most recent decade in all age groups (Fig. 1, Fig. 2 and 4 and Table 1). This is concerning, as HDP is still a major cause of maternal mortality, responsible for approximately 6·9% of the pregnancy-related deaths that occurred between 2011 and 2016 [17], [18], [19], as well as a major contributor to various severe maternal morbidities [3,17].

The reasons for the increasing trend in the prevalence of HDP are not well understood, but prior research has suggested increasing rates of co-morbidities, such as obesity, chronic hypertension (CH), diabetes mellitus (DM), advanced maternal age (AMA), and multiple gestations as potential explanations [10,20]. However, while we find that HDP is indeed more prevalent among vAMA women (Fig. 4), the long-term trend that we document in HDP behaves quite differently from the trends of CH, DM, and vAMA (Fig. 1, Fig. 2 and Table 1). Similarly, it does not correspond to the well-documented long-term continuous increase in obesity rates among pregnant women [21]. Therefore, while all of these suggested reasons may contribute to the increased prevalence of HDP, our results suggest that there may be additional contributing factors. Further research is needed to explore these potential factors.

The prevalence of CH rose dramatically since 1999, with an especially high APC during the last couple of years. Nowadays, CH, which affected only 0·65%−0·72% of pregnant women in the 1990s, has become significantly more common, with a prevalence of 2·07% in 2018 (Fig. 1, Fig. 2 and Table 1). Potential factors that may have contributed to this increase include increasing rates of obesity and AMA [15], and indeed, we find that the prevalence of CH among women with vAMA was more than double the overall prevalence. Nonetheless, higher relative increases in the rates of CH (i.e. higher AAPCs and RRs) among younger women during the past 30 years have made CH a noteworthy risk factor even among young women (Fig. 5).

Similarly, the prevalence of DM has more than doubled since the early 2000s, growing continuously at disturbing rates (Fig. 1, Fig. 2 and Table 1). Poor dietary habits, physical inactivity, as well as increasing rates of obesity and AMA, have been reported as potential contributors to this increase [10,16,[22], [23], [24], [25]]. General improvements in maternal screening and detection processes, as well as changes in diagnostic criteria and reporting practices may have also played a role [10,16,[22], [23], [24], [25]]. Part of the increase that we document can also be attributed to the 2003 revision of the US Standard Certificate of Live Birth, which was adopted in a staggered fashion across states until 2016, and has been found to increase the reported rates of DM [26,27]. In any case, in 2018, DM affected 7·61% of pregnant women.

DM among pregnant women is a known contributor to maternal morbidity and mortality [27,28]. In fact, Nelson et al. (2018) found that 17% of the increase in maternal mortality rates in the US between 1997 and 2012 was attributed to the increase in the prevalence of DM [27]. Of specific concern is that during the most recent decade, the prevalence of DM has grown among vAMA women at an especially high AAPC, so that an astounding 16·00% of vAMA women in 2018 were affected by DM (Fig. 6).

The prevalence of vAMA increased significantly over the last three decades, and primarily in the 1990s (Fig. 1, Fig. 2 and Table 1). In fact, over the last three decades, the proportion of pregnancies attributable to women in all of the age groups at or above the age of 30 increased, but the most notable increase was for vAMA women (whose proportion of pregnancies grew threefold, from 1.14% in 1989 to 3.35% in 2018; Fig. 3). This increase can be explained by the significant advancement in reproductive therapies during the 1990s and by the continuous trend of delaying family planning, which can potentially be attributed to improved access to education and contraception, as well as to an increased focus on the career development of women [29], [30], [31], [32]. Prior research has found that women at or above the age of 40 have higher rates of various pregnancy risk factors and complications, as well as an increased risk of maternal mortality [8,[29], [30], [31], [32]]. Our findings confirm these results. Specifically, we find that older women with vAMA consistently had a higher prevalence of HDP throughout the years (Fig. 4) and that the prevalence of both CH and DM consistently increased with age, peaking in the vAMA age group (Fig. 5, Fig. 6). Nonetheless, our study documents significant increases in the prevalence of HDP, CH, and DM across all age groups, and further solidifies their position as major risk factors/complications among all pregnant women in the US.

The overall increase in the prevalence of GM was relatively moderate during the past three decades, but practically all of this increase has occurred since 2006. In 2018, 5·38% of live births were attributed to grand multiparous women (Fig. 1, Fig. 2 and Table 1). The clinical significance of this finding is unclear due to inconsistent data on the risks associated with GM. While certain studies have found GM to be associated with various pregnancy complications [33], [34], [35], [36], [37], other studies, primarily in recent years, have suggested that in developed countries with satisfactory prenatal care, GM is not associated with additional risk [38], [39], [40].

However, it should be noted that significant racial and ethnic disparities exist with regard to all of these risk factors/complications. In a previous study, Bornstein et al. (2020), we reported that between 2007 and 2018 non-Hispanic Black women had the highest prevalence of HDP, CH, and GM, that Hispanic women had the highest prevalence of DM, and that non-Hispanic White women had the highest prevalence of AMA (which was defined as women at or above the age of 35) [7]. Additionally, we found that with the exception of GM, Hispanic women exhibited the highest increase in all of these risk factors/complications, followed by non-Hispanic Black women [7]. Table 2 provides a brief summary of our main results in Bornstein et al. (2020).

**Table 2:** Summary of Bornstein et al. (2020) – “Racial Disparity in Pregnancy Risks and Complications in the US: Temporal Changes during 2007–2018″ The table presents a summary of the results from Bornstein et al. (2020), which examined racial and ethnic disparities in the prevalence of hypertensive disorders of pregnancy (HDP), chronic hypertension (CH), diabetes mellitus (DM), very advanced maternal age (vAMA), and grand multiparity (GM), during the 2007–2018 time period. Changes in the prevalence between 2007 and 2018 are presented as odds ratios (OR).

	Non-Hispanic White Women	Non-Hispanic Black Women	Hispanic Women
**HDP**	2007 Prevalence: 4·4% 2018 Prevalence: 7·6% OR 1·75, 95% CI [1·74–1·76] *p*<0·001	2007 Prevalence: 4·6% 2018 Prevalence: 8·6% OR 1·94, 95% CI [1·91–1·97] *p*<0·001	2007 Prevalence: 2·8% 2018 Prevalence: 5·9% OR 2·23, 95% CI [2·20–2·26] *p*<0·001
**CH**	2007 Prevalence: 1·1% 2018 Prevalence: 1·9% OR 1·74, 95% CI [1·71–1·77] *p*<0·001	2007 Prevalence: 2·2% 2018 Prevalence: 4·0% OR 1·91, 95% CI [1·87–1·95] *p*<0·001	2007 Prevalence: 0·5% 2018 Prevalence: 1·4% OR 2·51, 95% CI [2·44–2·60] *p*<0·001
**DM**	2007 Prevalence: 4·2% 2018 Prevalence: 6·8% OR 1·61, 95% CI [1·59–1·62] *p*<0·001	2007 Prevalence: 3·9% 2018 Prevalence: 6·5% OR 1·71, 95% CI [1·68–1·73] *p*<0·001	2007 Prevalence: 4·6% 2018 Prevalence: 8·3% OR 1·88, 95% CI [1·85–1·90] *p*<0·001
**AMA**	2007 Prevalence: 15·9% 2018 Prevalence: 18·5% OR 1·16, 95% CI [1·16–1·17] *p*<0·001	2007 Prevalence: 10·2% 2018 Prevalence: 15·1% OR 1·57, 95% CI [1·55–1·59] *p*<0·001	2007 Prevalence: 11·0% 2018 Prevalence: 16·7% OR 1·62, 95% CI [1·61–1·64] *p*<0·001
**GM**	2007 Prevalence: 3·4% 2018 Prevalence: 4·4% OR 1·29, 95% CI [1·28–1·30] *p*<0·001	2007 Prevalence: 6·8% 2018 Prevalence: 8·1% OR 1·21, 95% CI [1·19–1·23] *p*<0·001	2007 Prevalence: 5·5% 2018 Prevalence: 6·4% OR 1·19, 95% CI [1·17–1·20] *p*<0·001

Our study has several strengths. In using the CDC natality files, which contain data on maternal risk factors/complications since 1989, we are able to explore the long-term trends over a period of three decades. Tracking trends over a long period of time can facilitate our understanding of the underlying causes for the increasing rates of maternal morbidity and mortality in the US, and in-turn contribute to improving the quality of maternal care. Additionally, the CDC database includes the entire population of US pregnant women with live births and is not limited by the characteristics of a specific institution or community. Thus, our study relies on the largest and most comprehensive dataset to evaluate the trends in these risk factors/complications.

Our study also has several limitations. As a large registry-based database, the CDC database includes registries with incomplete or missing records. It is also limited to live births, which can lead to underrepresentation of the most severe cases that have not resulted in a live birth. Moreover, the long-term trends of different hypertensive disorders of pregnancy or different types of diabetes cannot be assessed separately in the CDC database. Instead, gestational hypertension, preeclampsia, and HELLP syndrome are lumped under hypertensive disorders of pregnancy, and DM type I, DM type II and gestational DM are lumped under DM prior to the implementation of the 2003 revision of the US Standard Certificate of Live Birth. Finally, during the 30-year period between 1989 and 2018, several changes to maternal screening processes and data collection processes may have affected the reported rates of the risk factors/complications. The 2003 revision of the US Standard Certificate of Live Birth has been a meaningful change [26,27]. Other changes, such as new recommendations for gestational diabetes screening that came into effect in 2014, may have also had contributing effects. These changes need to be considered when interpreting our results.

In conclusion, we document significant ongoing increases in the prevalence of hypertensive disorders of pregnancy, chronic hypertension, diabetes mellitus, very advanced maternal age and grand mulitparity between 1989 and 2018. Our results provide evidence that the health of pregnant women in the US has worsened over the past three decades and that it continues to do so at alarming rates. Furthermore, our analysis indicates significantly increased risks associated with advanced maternal age and delaying childbearing. It could inform both clinicians and policy makers and help guide recommendations regarding the health of pregnant women, as well as highlight the importance of investing in improving young women's cardiovascular and metabolic health. Further research could be helpful in determining the causes for the increasing prevalence of these risk factors/complications and in evaluating their contribution to maternal morbidity and mortality.

## Contributors

EB was the principal investigator. EB, YE, FAC and AG conceived and designed the study. YE collected and analyzed the data, and wrote the initial draft of the manuscript. EB, FAC and AG were responsible for essential revisions of the manuscript and for the incorporation of important intellectual content. All authors reviewed the manuscript and gave final approval for submission.

## Funding

None.

## Data Sharing Statement

All of the data used in this study are publicly available from the National Center for Health Statistics.

## Declaration of Competing Interest

The authors report no conflicts of interest.
